# The SWI/SNF chromatin remodeling complex: a critical regulator of metabolism

**DOI:** 10.1042/BST20231141

**Published:** 2024-04-26

**Authors:** Michael C. Church, Jerry L. Workman

**Affiliations:** Stowers Institute of Medical Research, 1000 E 50th Street, Kansas City, MO 64118, U.S.A.

**Keywords:** chromatin, metabolism, transcription

## Abstract

The close relationship between chromatin and metabolism has been well-studied in recent years. Many metabolites have been found to be cofactors used to modify chromatin, and these modifications can in turn affect gene transcription. One chromatin-associated factor responsible for regulating transcription is the SWI/SNF complex, an ATP-dependent chromatin remodeler conserved throughout eukaryotes. SWI/SNF was originally described in yeast as regulating genes involved in carbon source metabolism and mating type switching, and its mammalian counterpart has been extensively studied for its role in diseases such as cancer. The yeast SWI/SNF complex is closely associated with activation of stress response genes, many of which have metabolic functions. It is now recognized that this is a conserved function of the complex, and recent work has shown that mammalian SWI/SNF is also a key regulator of metabolic transcription. Emerging evidence suggests that loss of SWI/SNF introduces vulnerabilities to cells due to this metabolic influence, and that this may present opportunities for treatment of SWI/SNF-deficient cancers.

## Introduction

Chromatin is the protein-DNA complex present in the nuclei of all eukaryotic cells, which functions both to package genomic data and dynamically influence gene expression. In addition to these canonical functions, in recent years it has been demonstrated that chromatin also has a role in metabolic homeostasis [1–4]. This is due to the properties of the basic repeating unit of chromatin, the nucleosome, which consists of a histone octamer wrapped in ∼147 bp of DNA [5]. These histones can be post-translationally modified with metabolites, and in this way chromatin may function as a ‘sink’ or storage compartment for cellular metabolites [1,3,4]. Incorporating metabolites into chromatin by post-translational modification (PTM) of histones can alter the properties of chromatin and affect gene transcription [6]. The commonly appreciated role for nucleosomes is as a DNA packaging mechanism that can influence gene expression. Nucleosome occupancy near transcription start sites is generally associated with inhibition of gene transcription, but this inhibition can be overcome or reinforced in many ways. In addition to PTM, nucleosomes can also be ‘remodeled’ by ATP-dependent remodeling complexes. A notable example of such a complex is SWI/SNF.

The SWI/SNF chromatin remodeling complex functions by displacing nucleosomes near important regulatory sites which can facilitate transcription factor binding and thus promote gene activation in both uni- and multicellular eukaryotes [7–15].SWI/SNF activity can be influenced by the types of modification(s) present on a nucleosome, that can either promote or inhibit nucleosome remodeling and transcription factor occupancy at particular genomic loci [16]. Chromatin remodeling by SWI/SNF is dependent on ATP, and SWI/SNF complexes can also recognize acetylated histones via bromodomains [17,18]. Both ATP and acetyl-CoA are required for ATPase activity and histone acetylation, respectively. These can be generated through carbohydrate/lipid metabolism, underscoring the relationship between chromatin, metabolism, and proteins that interact with both [19–21]. This complicated and interconnected relationship also offers opportunities for complexes such as SWI/SNF to sense nutritional status. For example, dynamic deacetylation of histones and reallocation of acetyl-CoA in response to starvation could affect SWI/SNF activity both through adding or removing acetylated histones recognized by the complex, and a reduction in cellular ATP levels required by SWI/SNF to remodel nucleosomes [22]. It has also been shown that the yeast SWI/SNF complex itself is acetylated, affecting its function in response to stress [23,24].

The SWI/SNF complex has emerged as an important regulator of gene expression in recent decades, with roles ranging from promoting cardiac and neuronal cell maturation to disease, where some SWI/SNF component is mutated in >20% of all cancers [15,25]. Although its utility as a target for therapy in humans is now well-recognized, initially the constituent subunits of this complex were described in the yeast *Saccharomyces cerevisiae*, and named for their inability to derepress genes involved in mating type switching (SWI) and carbon source metabolism (sucrose non-fermentable) [26,27]. Whole-genome studies in yeast subsequently showed that loss of SWI/SNF affected the expression of many other genes [28]. More recent work has shown that SWI/SNF can also co-operate with other, similar complexes to activate genes involved in amino acid metabolism [29,30].

Here, we review recent advancements in our understanding of the relationship between SWI/SNF and metabolism in several contexts, and how this relates to the earlier work done on complex functions. This review will primarily focus on carbon source (glucose), lipid and amino acid metabolism, and provide recent examples of the contribution of SWI/SNF complexes to metabolic regulation.

## History of SWI/SNF discovery: early metabolism links

**Figure 1.**
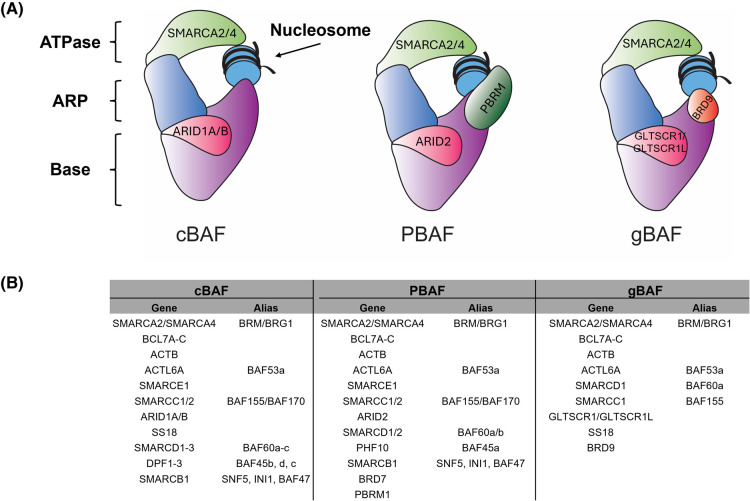
Complex subunits. (**A**) Schematic depicting canonical BAF (cBAF), polybromo BAF (PBAF) and non-canonical or GLTSCR1/like-containing BAF complex (ncBAF/gBAF). Different modules are colored differently, and some unique features are highlighted. **(B)** Table depicting three SWI/SNF complexes using information from [15], [39] and [40].

Some of the earliest work on SWI/SNF established the complex's involvement in carbon source metabolism [26]. This early work focused on mutants that were unable to express the *SUC2* gene, which encodes the enzyme invertase, the secreted form of which was found to be repressed by the presence of glucose [31]. This involvement in carbohydrate metabolism was recapitulated in subsequent years, and it was found that the mechanism by which SWI/SNF activated transcription of these genes in part by modifying promoter chromatin structure and facilitating activator binding [7,8,32–34]. These early mechanistic studies demonstrated that the SWI/SNF gene products formed a complex, and that this complex could modify chromatin and promote binding of the Gal4 transcription factor to nucleosomal DNA [7,8,35,36]. As Gal4 activates genes in response to glucose starvation, this was a direct link between chromatin remodeling activity and carbon source metabolism. Dynamic gene regulation mediated by chromatin remodelers such as SWI/SNF allows cells to switch between metabolic pathways in response to rapidly-changing stimuli. Although SWI/SNF has been most closely associated with glucose metabolism, emerging work has shown that this may only be part of its involvement in regulating metabolic processes [29,30,37,38]. The early studies had been mainly performed in yeast, but having identified the involvement of SWI/SNF in human disease, the logical next step was to investigate the mammalian complex. One complication when studying SWI/SNF complexes (sometimes called BAF complexes) in human cells is the sheer variety of their assemblies. There are many variants of the complex, including canonical BAF (cBAF), PBAF and non-canonical or ncBAF/gBAF among others, each distinguished by the presence of unique subunits (Figure 1) [15,41]. Despite this complexity, conservation of function in terms of regulation of metabolism can still be appreciated. Outlined below are some recent examples of studies supporting the role of SWI/SNF as an important regulator of gene expression in humans as well as yeast.

## Glucose metabolism

**Figure 2.**
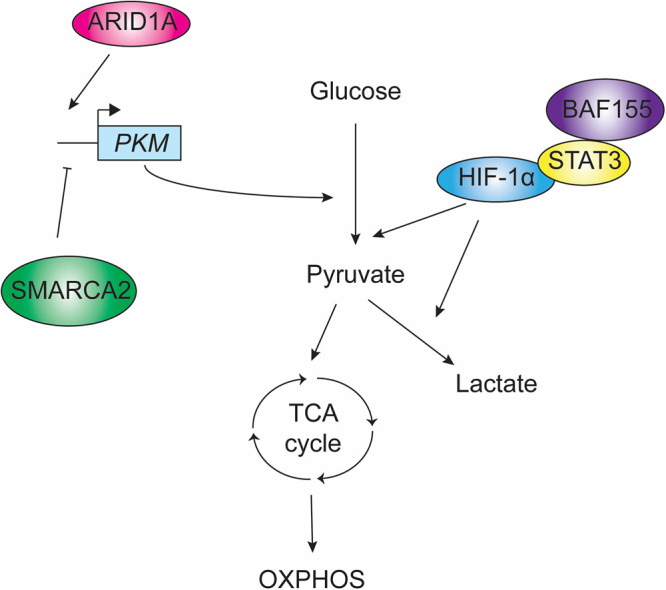
SWI/SNF and glucose metabolism. Schematic summarizing elements of energy generation from glucose, and highlighting parts of this pathway found to be affected by loss of SWI/SNF subunits: ARID1A was found to promote expression of *PKM* in a hepatocellular carcinoma model [48], whereas in a bladder cancer model, loss of SMARCA2 and other SWI/SNF subunits was found to increase PKM2 levels, suggesting that SWI/SNF is associated with *PKM* repression under these conditions [49]. During strenuous exercise, it was found that BAF155 associated with STAT3, which is required for HIF-1α-mediated gene expression [46]. HIF-1α is required for activation of genes involved in glycolysis and lactate production during hypoxia [50].

All cells require some form of carbon to generate energy for homeostasis. As discussed above, some of the earliest studies involving SWI/SNF described a role in regulation of genes involved in carbon source metabolism. Mammalian cells primarily rely on the TCA cycle for energy production, as it is the most efficient use of glucose in the presence of oxygen and can generate important signaling molecules used by the cell [42]. Under some conditions, such as during strenuous exercise, skeletal muscle cells can instead generate much of their energy anaerobically via glycolysis, which is also the method favored by yeast [43,44]. In humans, this is in part mediated by the hypoxia-induced transcription factor HIF-1α, as the energy requirement in muscle cells can outstrip the ability to acquire oxygen for respiration, resulting in a hypoxic environment [45]. One recent study has shown that SWI/SNF has a role in this process. In a mouse model, the core SWI/SNF subunit BAF155 (SMARCC1) was found to be dispensable for development and growth of skeletal muscle, but loss of BAF155 resulted in enhanced endurance exercise capacity [46]. The mechanism behind this phenotype became clearer when it was found that SWI/SNF and the STAT3 protein physically interact, and loss of BAF155 abolished this interaction. STAT3 and HIF-1α co-operate to activate HIF-1α target genes, and SWI/SNF mutants were unable to perform this function [46,47]. This biased glucose metabolism away from glycolysis and towards the TCA cycle, improving the efficiency of ATP generation, and resulted in improved skeletal muscle endurance (Figure 2) [46].

In addition to the central role that carbon source metabolism plays in healthy cells, it has been known for some time that tumor cells exhibit perturbations in glucose metabolism, neglecting the TCA cycle in favor of glycolysis and lactic acid production to generate energy [51,52]. This makes understanding the regulation of these processes especially important. Interestingly, there have been several recent examples of SWI/SNF-deficient cancers which actually have a strong reliance on the TCA cycle instead of lactic acid biosynthesis. In one study which used a lung adenocarcinoma model, the SWI/SNF ATPase SMARCA4 (BRG1) was found to be a tumor suppressor, largely due to its impact on glucose metabolism [53]. It was discovered that SMARCA4-deficient cells displayed increased mitochondrial respiration, and inhibiting oxidative phosphorylation in glucose-starved, SMARCA4-deficient cells led to cell death due to the cells’ inability to properly activate genes involved in energy stress, including genes required for stimulation of glycolysis such as HIF2A [53]. This is not the only example of SWI/SNF-deficient cancers showing a reliance on mitochondrial respiration. Another study in a hepatocellular carcinoma model investigated metabolic perturbations in tumor cells with mutations in the ARID1A (BAF250) subunit of SWI/SNF [48]. It was found that ARID1A-deficient cells showed reduced transcription of glycolytic enzyme-encoding genes, leaving these cells more reliant on energy generation via the TCA cycle. This led to increased mitochondrial respiration and rendered ARID1A-deficient tumor cells vulnerable to drugs that target the TCA cycle and/or oxidative phosphorylation [48]. These findings were promising, but due to the complexity of SWI/SNF function and unpredictable consequences of loss of this important chromatin regulator, the effect of SWI/SNF loss may have differing effects depending on cell type. One study revealed that in patient-derived bladder cancer tumors, dysregulation of several SWI/SNF subunits (SMARCA2, INI1 and BAF155) again correlated with alterations in expression of genes involved in glucose metabolism [49]. However, unlike the previous two studies carried out in different cell types, this study found that low expression of the ATPase SMARCA2 (BRM) was found to increase expression of the glycolytic enzyme pyruvate kinase M2 (PKM2), and this correlated with bladder cancer metastasis [54]. Although these findings highlight apparently contradictory SWI/SNF functions (promoting vs repressing glycolysis), they may instead emphasize the important role the complex plays in overseeing the delicate balance of carbon source metabolic priorities.

The mechanisms by which SWI/SNF senses and responds to metabolic stimuli are less clear, but new insights are emerging. For example, a recent study performed in *S. cerevisiae* probed the mechanism by which the SWI/SNF complex senses carbon source starvation which may also shed light on processes occurring in mammalian cells [55]. It was already known that transient, intracellular acidification is correlated with transcriptional regulation of some stress response genes [56]. This latest study proposed that disordered, glutamine-rich regions of the Snf5 subunit of SWI/SNF responded to these pH changes. The authors hypothesized that Snf5 regulated transcription of carbon source genes (including those coding for part of the TCA cycle) by affecting interactions with activating transcription factors in a pH-dependent manner. Changing pH may impact the conformation of these glutamine-rich regions presenting opportunities for novel protein interactions, directly linking nutritional status to transcription via intracellular conditions [55]. Similar to Snf5, the Swi1 subunit possesses a glutamine and asparagine-rich N-terminus, with the latter protein being reported to form a prion [57]. These examples underscore the complexity of the intersection between chromatin remodeling and metabolism and emphasize the importance of understanding these processes at a fundamental level. It also highlights the conservation between yeast and human metabolic pathways.

## Lipid metabolism

Chromatin, glucose, and lipid metabolism are closely interlinked because a carbohydrate-rich diet stimulates lipogenesis, and fatty acids can also be oxidized to yield acetyl-CoA [19,21]. A role for SWI/SNF at the interface between glucose and lipid metabolism was recently described, implicating the ARID1A subunit in this process [58]. In a mouse model, hepatic knockdown of ARID1A was associated with a defect in fatty acid oxidation leading to lipid accumulation in the liver. This correlated with decreased glucose tolerance/insulin sensitivity and elevated weight gain when mice were fed a high fat diet. The cause of this defect was proposed to be a down-regulation of genes involved in fatty acid oxidation in the liver. One of the fatty acid oxidation genes down-regulated in cells lacking ARID1A encoded PPARα, which is a transcription factor important for stimulating fatty acid oxidation [59]. Down-regulation of PPARα in turn affected its target genes, ultimately leading to lipid accumulation and insulin resistance [58]. In addition to a role for ARID1A in regulating fatty acid oxidation in the liver, another SWI/SNF subunit, BRG1, has been implicated in the promotion of lipid synthesis in a breast cancer model [60,61].

It has been noted that BAF60c (SMARCD3) has a role in regulation of glucose metabolism [62]. Therefore, due to the close relationship between these pathways it is perhaps not surprising that another BAF60 isoform, BAF60a (SMARCD1), has a role in lipid metabolism, being highly expressed in the brain, liver and adipose tissues [63]. In macrophages, down-regulation of BAF60a has been associated with in the onset of atherosclerosis [64]. Macrophages are often important drivers of atherosclerosis due to their role in inflammation, which is thought to contribute to the onset of the disorder [65,66] It was found that in a mouse model, BAF60a was down-regulated in plaque macrophages in advanced atherosclerosis, and generation of BAF60a-deficient macrophages resulted in mitochondrial dysfunction, which is associated with inflammation [67]. The BAF60 subunits are known to interact with transcription factors, and reduced BAF60a expression was found to correlate with impaired activity of the mitochondria-associated transcription factor NRF1, likely explaining the mitochondrial dysfunction in macrophages correlated with atherosclerosis [64]. Although BAF60c is strongly associated with regulation of glucose metabolism, a study carried out in fish found a role for this isoform in lipid metabolism. In the economically-relevant large yellow croaker (*Larimichthys crocea*), it was found that BAF60c interacted with and promoted expression of the heat shock-related protein GRP78 [68]. Furthermore, it was found that BAF60c and GRP78 co-operated to promote lipogenesis, ER stress, and inflammation, providing more evidence of the central role that SWI/SNF plays in these processes [68].

Although as unicellular organisms, yeast do not have the same signaling and lipid storage mechanisms as mammals, there is still evidence that regulation of lipid metabolism is a conserved function of SWI/SNF in these species. For example, several SWI/SNF subunits were identified in a screen of factors involved in lipid droplet consumption [69]. There is also evidence that SWI/SNF regulates genes important for phospholipid biosynthesis in *S. cerevisiae* [70]. Phospholipid biosynthesis is closely associated with other metabolic processes, as the generation of phospholipids is the largest consumer of *S*-adenosyl methionine (SAM) in *S. cerevisiae* [3]. SAM is produced by the sulfur metabolism pathway, which is another area of metabolism recently found to be affected by SWI/SNF.

## Amino acid metabolism

**Figure 3.**
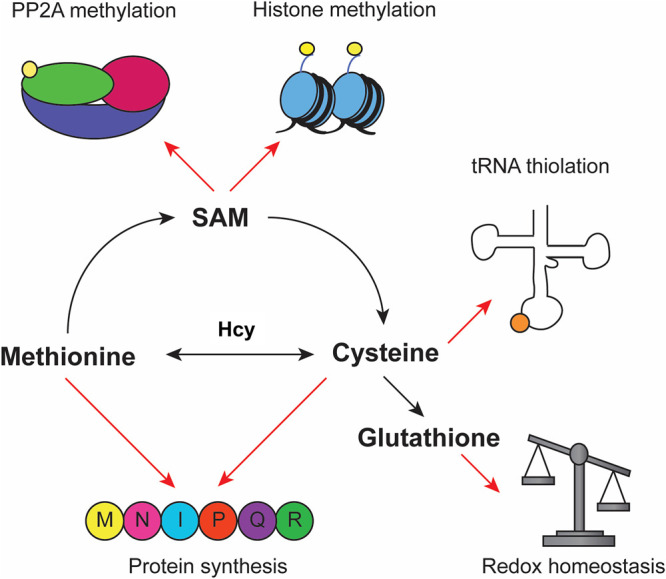
Sulfur metabolism and its products. Schematic depicting some of the products of sulfur metabolism. Dietary methionine is incorporated into peptides or to synthesize *S*-adenosyl methionine (SAM), which is a cofactor required for methylation reactions [73,74]. SAM is converted to homocysteine (Hcy), which can be used to synthesize cysteine. Cysteine can be incorporated into peptides, but is also a cofactor required for tRNA thiolation, and is required to synthesize glutathione [75,76].

Much like glucose and lipid metabolism, regulation of amino acid biosynthesis is not only critical for cellular homeostasis, but also plays a central role in disease. While numerous studies have investigated the role of yeast SWI/SNF in different aspects of amino acid metabolic gene transcription, several recent examples focus on the sulfur-containing amino acids methionine and cysteine [23,28–30,37]. In addition to carbon source metabolic rewiring, tumor cells also alter their sulfur/methionine metabolism, likely to compensate for aberrant methylation reactions occurring in these cells [71]. These pathways are complex and interlinked, and understanding their regulation is critical for identifying targets for treatments in disease. As with carbon source utilization, yeast provided the first evidence of SWI/SNF involvement in amino acid metabolism, though the effects were more complicated than repression of glucose-repressed genes. The first whole-transcriptome study on *snfΔ* mutants identified elevated transcription of genes involved in sulfur and serine metabolism [28]. In the case of the latter (*SER3*), it was found that SWI/SNF activated an intergenic transcript (*SRG1*) in growth under *SER3*-repressing conditions and that transcription of *SRG1* interfered with *SER3* expression [72]. The mechanism by which SWI/SNF loss affected sulfur metabolism was less clear, but it was recently demonstrated that during the growth in the presence of sulfur (repressing conditions), SWI/SNF was required for transcription of genes involved in cysteine biosynthesis. Loss of SWI/SNF under these conditions resulted in a starvation-like response and activation of many genes involved in sulfur metabolism [38]. Sulfur-containing metabolites have many important roles in homeostasis and disease, making these pathways an important focus for medical research (Figure 3).

Although the findings that SWI/SNF affected sulfur metabolism were initially made in yeast, once again they foreshadowed discoveries made in human cells with relevance for disease. One example concerns ARID1A-deficient ovarian cancers, where it was found that SWI/SNF was required for activation of the *SLC7A11*-encoded cysteine transporter [77]. Cysteine is required for the synthesis of glutathione, the reduced form of which (GSH) is critical for redox homeostasis [78]. These tumor cells were deficient in GSH and therefore vulnerable to treatment with an agent that inhibited GSH synthesis and caused the cells to succumb to oxidative stress [77]. As ARID1A mutations are found in many cancers and associated with poor prognoses, this represents a promising future therapy. This is not the only case of SWI/SNF complexes modulating cysteine metabolism in a disease context. Another example of the involvement of SWI/SNF in regulation of sulfur amino acid metabolism concerns the gBAF complex. Mutation of gBAF component BRD9 is associated with increased risk of cutaneous melanoma. A recent study has shown that a point mutation in BRD9 affected cysteine metabolism in A375 cells. This led to a correlation between disruption of cysteine metabolism and development and aggressiveness of melanoma [79]. The involvement of SWI/SNF complexes in regulating sulfur metabolism is of particular interest because of the aberrant methylation reactions that occur within many tumor cells, as a sulfur-containing metabolite (*S*-adenosyl methionine/SAM) is a required cofactor for these reactions [71]. Additionally, due to the elevated levels of oxidative stress borne by tumor cells, these cells become dependent on the otherwise non-essential amino acid cysteine [80]. In a non-disease study performed on bovine cells, it was found that growth in the presence of methionine correlated with increased degradation and reduced nuclear localization of ARID1A and stimulation of genes involved in milk fat synthesis [81]. This further highlights the link between amino acid and lipid metabolism, and the role of SWI/SNF in regulating both processes.

Another study in yeast provides evidence for a direct link between sulfur metabolism and SWI/SNF. The Snf2 ATPase is a target of the methyltransferase Hmt1, and it was found that loss of Hmt1 catalytic activity correlated with reduced Snf2 promoter nucleosome remodeling [82]. The conclusion from this study was that Hmt1 expression is reduced when cells encounter stress allowing for more transcriptional stochasticity and therefore the potential for population survival. However as the cofactor required for methylation by Hmt1 is a direct product of sulfur metabolism, it is intriguing to speculate that sulfur starvation may also affect SWI/SNF PTM and function.

## Conclusions

SWI/SNF has emerged as a significant factor in human disease in recent years, with many cancers containing mutations in SWI/SNF subunits [15,25]. Mutation of regulatory factors such as SWI/SNF presents challenges for developing effective therapies, as loss of such factors can lead to widespread, complicated changes in cellular physiology. However, the role of SWI/SNF as a metabolic regulator reveals vulnerabilities of tumor cells, which may be exploitable [48,77]. Here, we have highlighted the involvement of SWI/SNF in regulation of glucose, lipid and amino acid metabolism. In several examples, mammalian SWI/SNF was shown to function in a similar fashion to the yeast complex, promoting the expression of stress response genes and activating alternative metabolic pathways. When these pathways were inhibited, cells became more susceptible to treatment. Identifying synthetic lethality is a good way to combat loss-of-function mutations in tumor suppressors such as SWI/SNF, and this requires a deeper understanding of the basic biology of metabolic pathway regulation [48].

**Figure 4.**
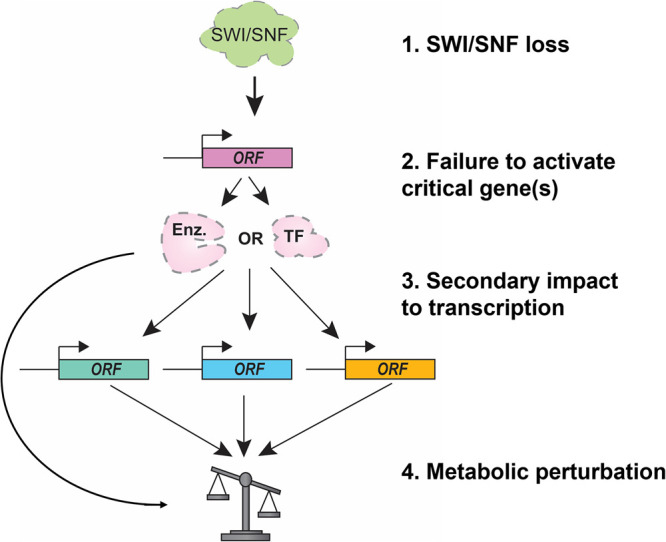
Indirect impact of SWI/SNF loss on metabolic pathways. Model for indirect impact of SWI/SNF loss on metabolic pathways. (**1**) Loss of SWI/SNF affects transcription of its direct target genes. (**2**) If direct targets of SWI/SNF encode transcription factors (TF) or enzymes (Enz.), the activities carried out by these gene products are reduced or abolished. (**3**) Loss of transcription factor activity or failure to synthesize an affected enzyme's product causes alteration in transcription of genes that respond to these signals. (**4**) As a result of this latest transcriptional impact, other enzyme-encoding genes are aberrantly repressed or activated, leading to metabolic perturbations.

A common theme with the metabolic impact of SWI/SNF loss is the widespread indirect effects that proceed from disruptions of direct SWI/SNF targets (Figure 4). Loss of transcription of a gene encoding a transcription factor has downstream implications for that protein's targets (e.g. PPARα and GRP78) [58,68]. Similarly, altered expression of an enzyme forming part of a metabolic pathway can lead to disturbed nutrient levels and subsequently elicit a stress response [38,48,49].

As outlined above, Swi-Snf was first described in the yeast *S. cerevisiae*. Although technological improvements have allowed more accessible investigations into human SWI/SNF complexes in recent years, yeast remains an attractive model for the study of metabolic pathways owing to its relative simplicity. The yeast SWI/SNF complex is less abundant than other related remodelers such as RSC, and under normal laboratory growth conditions most mutants are viable [83]. Rather than diminishing the importance of SWI/SNF, this creates opportunities for the cell to tune metabolic gene regulation in response to metabolic stimuli in a way that would not be possible with an essential complex. Perhaps this offers insight into the early evolution of the multiple, more specialized SWI/SNF complexes present in higher eukaryotes. Indeed, there is a remarkable amount of functional conservation between yeast and human complexes, and insights gained in yeast continue to inform work being undertaken to advance our understanding of the role of the complex in human health and disease.

## Perspectives

- SWI/SNF was the first chromatin remodeling complex to be characterized, initially in yeast, and then in multicellular eukaryotes. There are multiple SWI/SNF-like complexes found in humans, and SWI/SNF mutations are strongly associated with several types of cancer. Tumor cells have characteristic transcriptional and metabolic perturbations, and understanding the factors that influence gene expression and metabolic regulation is therefore critical for developing treatments.
- SWI/SNF has long been known as an activator of gene transcription, and early studies described a role for the complex in glucose metabolism in yeast. However, lately SWI/SNF has also emerged as an important regulator of metabolic genes in humans. Several recent studies have demonstrated that its role in cancer is in part related to regulation of metabolism.
- The discovery of vulnerabilities of SWI/SNF-deficient cancers relating to their effect on metabolism has shown potential therapeutic opportunities, and this may add to treatment options for more cancers in coming years. Our understanding of SWI/SNF as a metabolic regulator continues to evolve, and future studies may shed light on the mechanisms involved in metabolic sensing by SWI/SNF complexes.

## Abbreviations

cBAF: canonical BAF
PKM2: pyruvate kinase M2
PTM: post-translational modification
SAM: *S*-adenosyl methionine
